# *Doublecortin-like kinase* is required for cnidocyte development in *Nematostella vectensis*

**DOI:** 10.1186/s13064-024-00188-0

**Published:** 2024-06-22

**Authors:** Johanna E. M. Kraus, Henriette Busengdal, Yulia Kraus, Harald Hausen, Fabian Rentzsch

**Affiliations:** 1 Michael Sars Centre, University of Bergen, Thormøhlensgt 55, Bergen, 5006 Norway; 2 Department of Evolutionary Biology, Biological Faculty, Moscow State University, Leninskiye gory 1/12, Moscow, 119234 Russia; 3 Department for Biological Sciences, University of Bergen, Thormøhlensgate 53, Bergen, 5006 Norway; 4 Present Address: Department of Earth Science, University of Bergen, Allégaten 41, Bergen, 5007 Norway

**Keywords:** Neurite, Microtubule, Cytoskeleton, Cnidocyte, Nervous system evolution, Cnidaria, Doublecortin, Transgene, Dclk, Elav

## Abstract

The complex morphology of neurons requires precise control of their microtubule cytoskeleton. This is achieved by microtubule-associated proteins (MAPs) that regulate the assembly and stability of microtubules, and transport of molecules and vesicles along them. While many of these MAPs function in all cells, some are specifically or predominantly involved in regulating microtubules in neurons. Here we use the sea anemone *Nematostella vectensis* as a model organism to provide new insights into the early evolution of neural microtubule regulation. As a cnidarian, *Nematostella* belongs to an outgroup to all bilaterians and thus occupies an informative phylogenetic position for reconstructing the evolution of nervous system development. We identified an ortholog of the microtubule-binding protein doublecortin-like kinase (*NvDclk1*) as a gene that is predominantly expressed in neurons and cnidocytes (stinging cells), two classes of cells belonging to the neural lineage in cnidarians. A transgenic *NvDclk1* reporter line revealed an elaborate network of neurite-like processes emerging from cnidocytes in the tentacles and the body column. A transgene expressing NvDclk1 under the control of the *NvDclk1* promoter suggests that NvDclk1 localizes to microtubules and therefore likely functions as a microtubule-binding protein. Further, we generated a mutant for *NvDclk1* using CRISPR/Cas9 and show that the mutants fail to generate mature cnidocytes. Our results support the hypothesis that the elaboration of programs for microtubule regulation occurred early in the evolution of nervous systems.

**Supplementary Information:**

The online version contains supplementary material available at 10.1186/s13064-024-00188-0.

## Introduction

The elaborate morphology of neural cells is accompanied and enabled by proteins that regulate the assembly and dynamics of the different filaments that constitute the neural cytoskeleton. This can occur via direct stabilization/destabilization of cytoskeletal filaments, their posttranslational modification, or the binding to other molecules and cellular structures [1–6]. Microtubule-associated proteins (MAPs), for example, are important for the outgrowth and stability of neurites, for axonal and dendritic transport and for neuronal migration. Some MAPs appear to function specifically in neurons, whereas others have more generic roles in regulating microtubules in all cells [6–8]. While neural-specific MAPs have been identified and studied in vertebrates and a few other bilaterians, it is not well understood when during the evolution of nervous systems, they acquired their neural functions. Here we attempt to improve the understanding of the early evolution of neurons by studying the neural microtubule-associated protein Doublecortin-like kinase (Dclk) in the sea anemone *Nematostella vectensis.*

Doublecortin-like kinases are members of a small family of proteins characterized by the presence of one or two microtubule-binding doublecortin domains [9–11]. The name of this domain derives from the phenotype caused by mutation of the founding member of this protein family, *doublecortin* or *DCX* [12, 13]. Females heterozygous for mutations of this X-linked gene display a developmental defect of the cerebral cortex called subcortical band heterotopia (also described as double cortex syndrome), whereas males develop lissencephaly, a reduced folding of the cortex [12, 13]. These phenotypes reflect defects in the lamination of the cortex and are caused by impairment of neural migration required for the correct formation of the cortical cell layers [14, 15]. In contrast to the situation in humans, mutation of the *Dcx* gene in mice does not lead to an obvious phenotype in the cortex, though these mice display disorganization of the hippocampus [16]. Simultaneous knock-out of mouse *Dcx* and *Dclk1* resulted, however, in neuronal migration and cortical lamination defects resembling those observed in humans carrying *DCX* mutations [17–19]. The three mammalian Dclks differ from Dcx by the presence of a C-terminal serine/threonine kinase domain, but these findings suggest that *Dcx* and *Dclk1* have redundant functions in the control of neuronal migration during cortex development in mice. In addition to their role in neuronal migration, Dcx and Dclk1 have been shown to function in the formation of axons and dendrites [17, 18, 20–23]. These roles in neuronal migration and neurite formation have been attributed to the ability of Dcx and Dclk1 to bind microtubules, regulate their polymerization and affect the function of other microtubule-binding proteins [10, 14, 15, 20, 23–29]. Dcx and Dclk1/2 have also been shown to interact with actin filaments and this property has been proposed to contribute to the regulation of axon guidance [28, 30–32]. Outside the nervous system, *dcx* is expressed in muscle cells [33, 34] and Dclk1 has attracted considerable attention as a marker for tumour stem cells in a variety of cancers [35, 36].

Outside vertebrates, little is known about the role of Dcx and Dclk genes. A *Dcx* gene has been identified in the amoebozoan *Dictyostelium discoideum*, where it has a role in the aggregation of cells during development [37]. In *Drosophila melanogaster*, a gene encoding two dcx domains and multiple C-terminal WD40 domains (DCX-EMAP) is involved in the development and function of mechanosensory cells [38, 39], but no specific function has been identified for the Dclk gene *CG17528* [40]. In *Caenorhabditis elegans*, the Dclk gene *zyg-8* is required for microtubule assembly at the mitotic spindle of zygotes [41] and later during development as a regulator of microtubules mainly in neurons [42]. In sea urchin, two Dclk genes are expressed uniformly throughout the embryo [43]. Microtubule-binding Dcx domains thus emerged prior to the origin of metazoans, but it remains unclear at which point in evolution Dcx-domain proteins became important for the development of neural cells.

We identified a Dclk gene (*NvDclk1*) from the sea anemone *Nematostella vectensis* in a screen for genes whose expression is altered in conditions of increased or decreased neurogenesis (G. Richards, J. Blommaert, F. Rentzsch, unpublished data). *Nematostella* belongs to the cnidarians (e.g. corals, jellyfish, sea anemones) which are the sister group to the bilaterians (e.g. vertebrates, arthropods, nematodes) with a divergence time > 600 million years ago [44–46]. Cnidarian polyps are sessile, tube-shaped animals with a single body opening that is surrounded by tentacles for the capture of prey. Due to simple culture conditions and a relatively well-developed suite of tools for genetic manipulations, *Nematostella vectensis* has emerged as an important cnidarian model organism [47, 48]. Development in *Nematostella* includes a hollow blastula stage, gastrulation by invagination and a free-swimming planula stage before the animals transform into sessile and feeding primary polyps [47, 49, 50]. Neurogenesis occurs throughout the body column in tissues derived from both ectoderm and endoderm (the only two germ layers in cnidarians). It commences at blastula stage with the appearance of progenitor cells that give rise to secretory cells and the main classes of cnidarian neural cells, i.e. sensory/sensory-motor neurons, ganglion neurons (morphologically resembling interneurons) and the cnidarian-specific cnidocytes (stinging cells) [51–56]. The genetic programs controlling neurogenesis in *Nematostella* and in diverse bilaterians share many signalling molecules and transcription factors [48, 57–63], but how molecules involved in the elaboration of neuronal morphology and subcellular structures compare between cnidarians and bilaterians, is not well understood.

Using expression analyses and transgenic reporter lines, we show here that *NvDclk1* is expressed in neurons and cnidocytes and that the NvDclk1 protein is associated with microtubules in cnidocytes. The transgenic lines reveal a dense network of neurite-like processes emerging from cnidocytes in the tentacles. We also generated a *NvDclk1* mutant line via CRISPR/Cas9 and found that these mutants display defects in the development of the extrusive capsule of the cnidocytes. These observations support the hypothesis that regulation of microtubules by doublecortin-domain proteins is an ancient feature of nervous system development.

## Results

### *NvDclk1* is expressed in scattered cells from blastula stage on

**Fig. 1:**
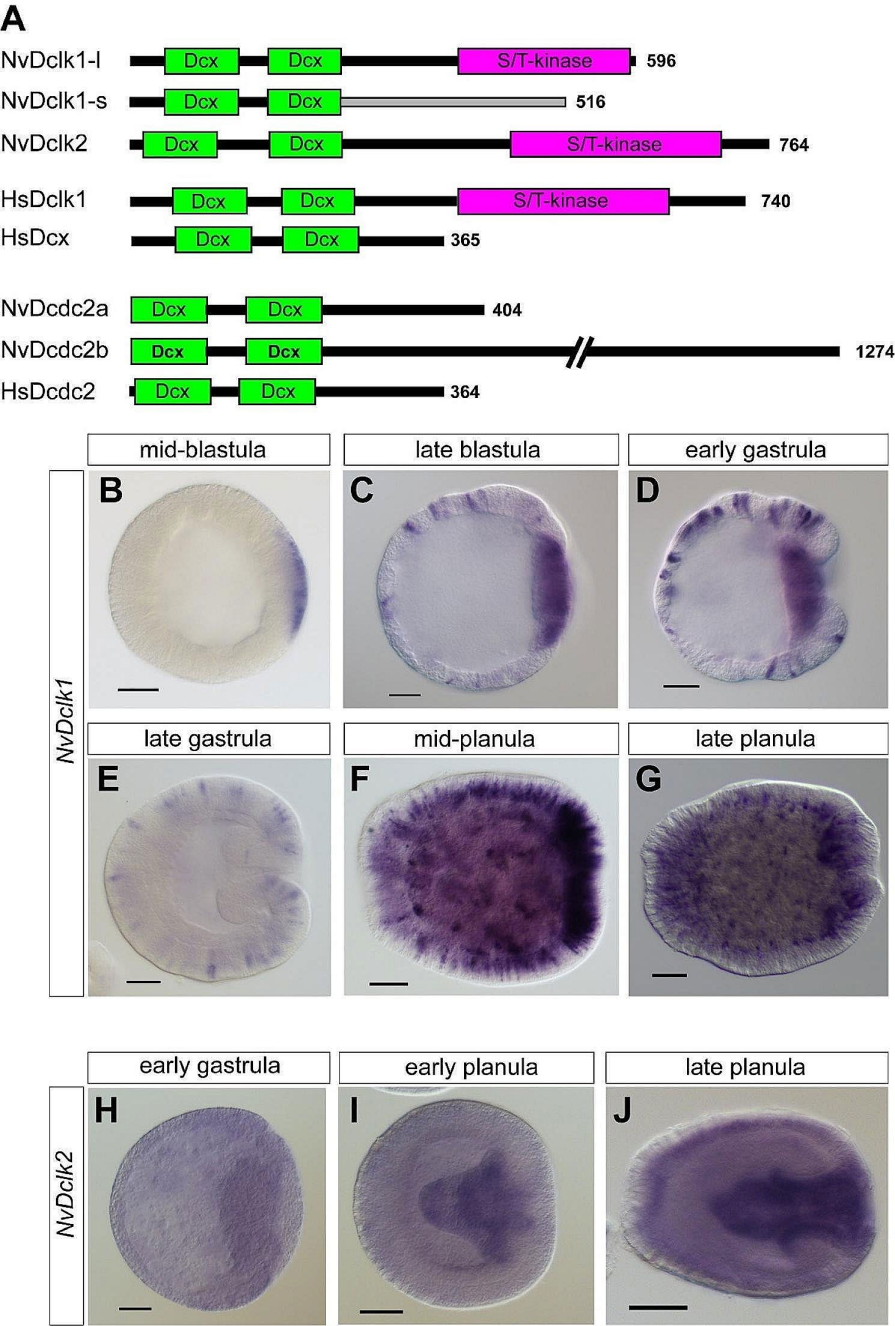
*NvDclk1* is expressed in scattered cells during development. (**A**) Schematic depictions of the domain composition of selected *Nematostella* (Nv) and human (Hs) dcx domain-containing proteins. The number of amino acids is indicated on the right side. (**B**-**J**) Lateral views of in situ hybridization with the oral pole oriented to the right. Probes are indicated on the left, developmental stages on top of the images. Note that for (**B**) the orientation is deduced from the expression in the endodermal plate at later stages. Scale bars: 50 μm

We identified two doublecortin-like kinase genes in the *Nematostella* genome, which we termed *NvDclk1* and *NvDclk2*. Both contain two dcx domains, typical of the Dclk1/2 subgroup and different from most Dclk3s that possess only one dcx domain [9, 64]. Two additional predicted genes encoding two N-terminal dcx domain, but no kinase domain, were most similar to vertebrate Dcdc2 genes in BLAST searches (Fig. 1A [9, 11]) . For *NvDclk1*, we cloned two splice variants, one encoding the dcx and kinase domains (*NvDclk1- long* or *NvDclk1l)*; and one that lacks the kinase domain (*NvDclk-short* or *NvDclk1s*, Fig. 1A). *NvDclk1s* is not a truncated version of *NvDclk1l*, instead the entire sequence after the second dcx domain is derived from exons that are not present in the mature *NvDclk1l* transcripts (Fig. 1A). Using a probe that recognizes both splice variants, we found that *NvDclk1* is first detectable at mid-blastula stage in a patch of tissue (Fig. 1B). At late blastula stage, this tissue thickens, identifying it as the future oral region that will give rise to the endoderm (Fig. 1C [49, 50]) . In addition, expression becomes visible in individual cells distributed throughout the blastoderm (Fig. 1C). The number of these cells increases during gastrulation, whereas the expression in the invaginating endoderm ceases (Fig. 1D, E). During planula stages, expression of *NvDclk1* remains detectable in the scattered ectodermal and endodermal cells of the body column and in particular around the oral pole, the site where the tentacles with a high density of cnidocytes form (Fig. 1F, G). *NvDclk2* is expressed throughout the *Nematostella* tissue, with higher levels in the pharynx during planula stages (Fig. 1H-J). Since the expression pattern of *NvDclk1* resembles the distribution of neural cells, we focused our subsequent analyses on this gene.

### A ***NvDclk1***::***GFP*** reporter line labels cnidocytes and neurons

**Fig. 2:**
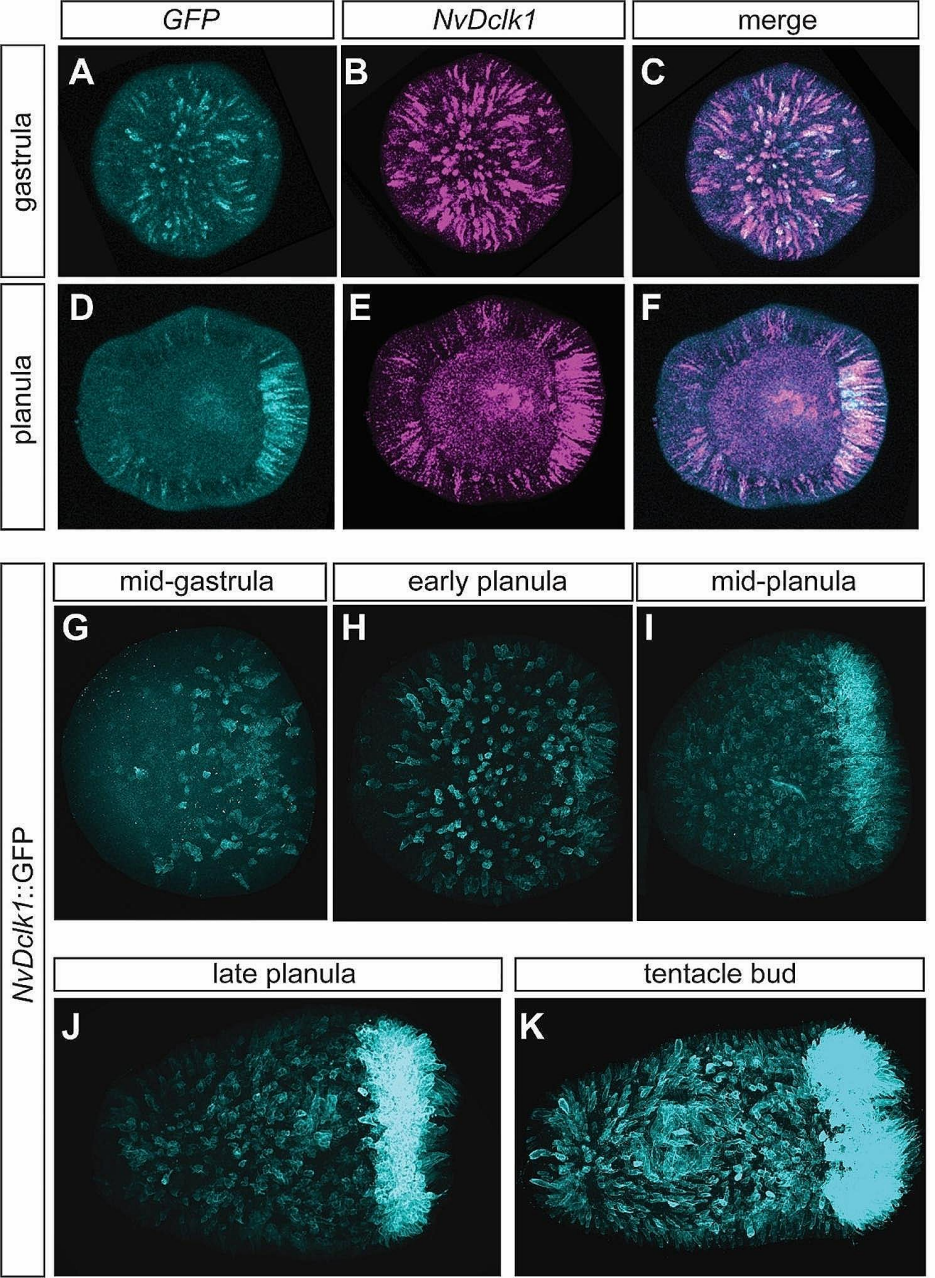
A *NvDclk1::GFP* reporter line labels a subset of *NvDclk1*-expressing cells. (**A**-**F**) Lateral views of double fluorescence in situ hybridizations, oral pole oriented to the right. Developmental stages are indicated on the left, probe on top. Not all *NvDclk1*-expressing cells also express the transgene-derived *GFP* mRNA. (**G**-**K**) Lateral views of *NvDclk1*::GFP transgenic animals at different developmental stages. GFP expression is detected by immunofluorescence with an anti-GFP antibody

To gain better insight into a potential expression of *NvDclk1* in neural cells, we first analysed existing transcriptome data. *NvDclk1* transcripts are enriched in transcriptomes derived from *NvElav1*::mOrange^+^ neurons (3.4 fold) and from *NvNCol3*::mOrange2^+^ cnidocytes (2.1 fold), both generated at primary polyp stage [61, 63, 65, 66]. Next, we generated a transgenic reporter line in which a 2.9 kb genomic DNA fragment immediately upstream of the *NvDclk1* start codon drives the expression of a membrane-tethered green fluorescent protein (GFP). We used double fluorescence in situ hybridization (DFISH) in this *NvDclk1::GFP* transgenic line to assess how well the line represents *NvDclk1* expression. Nearly all *GFP* expressing cells were also expressing *NvDclk1* (97.5 ± 0.6% at gastrula, 97.9 ± 3.6% at planula stage, *n* = 3 for each stage), but we also observed many *NvDclk1* expressing cells that did not express *GFP* (32.4 ± 5% of all *NvDclk1*-expressing cells at gastrula, 42 ± 17.4% at planula stage, Fig. 2A-F). The transgenic line can thus be used for tracing *NvDclk1*-expressing cells, though it does not identify all of these cells and their progeny. GFP-positive cells were detectable from gastrula stage on (Fig. 2G) and remained visible throughout the body column at planula and tentacle bud stages (Fig. 2H-K). From mid-planula stage on, strong GFP signal was visible at the oral end, in the developing tentacles (Fig. 2I-K). While the signal in the tentacles was readily visible in animals derived from crosses of *NvDclk1::GFP* and wildtype animals, the signal in the body column was clearly visible only in a fraction of planulae and

polyps derived from incrosses of *NvDclk1::GFP* animals. We assume that animals with visible signal in the body column contain two alleles of the transgene. Notably, we did not observe GFP expression in the pre-endodermal plate and the invaginating endoderm, despite clear signal in the *NvDclk1* in situ hybridizations.

**Fig. 3:**
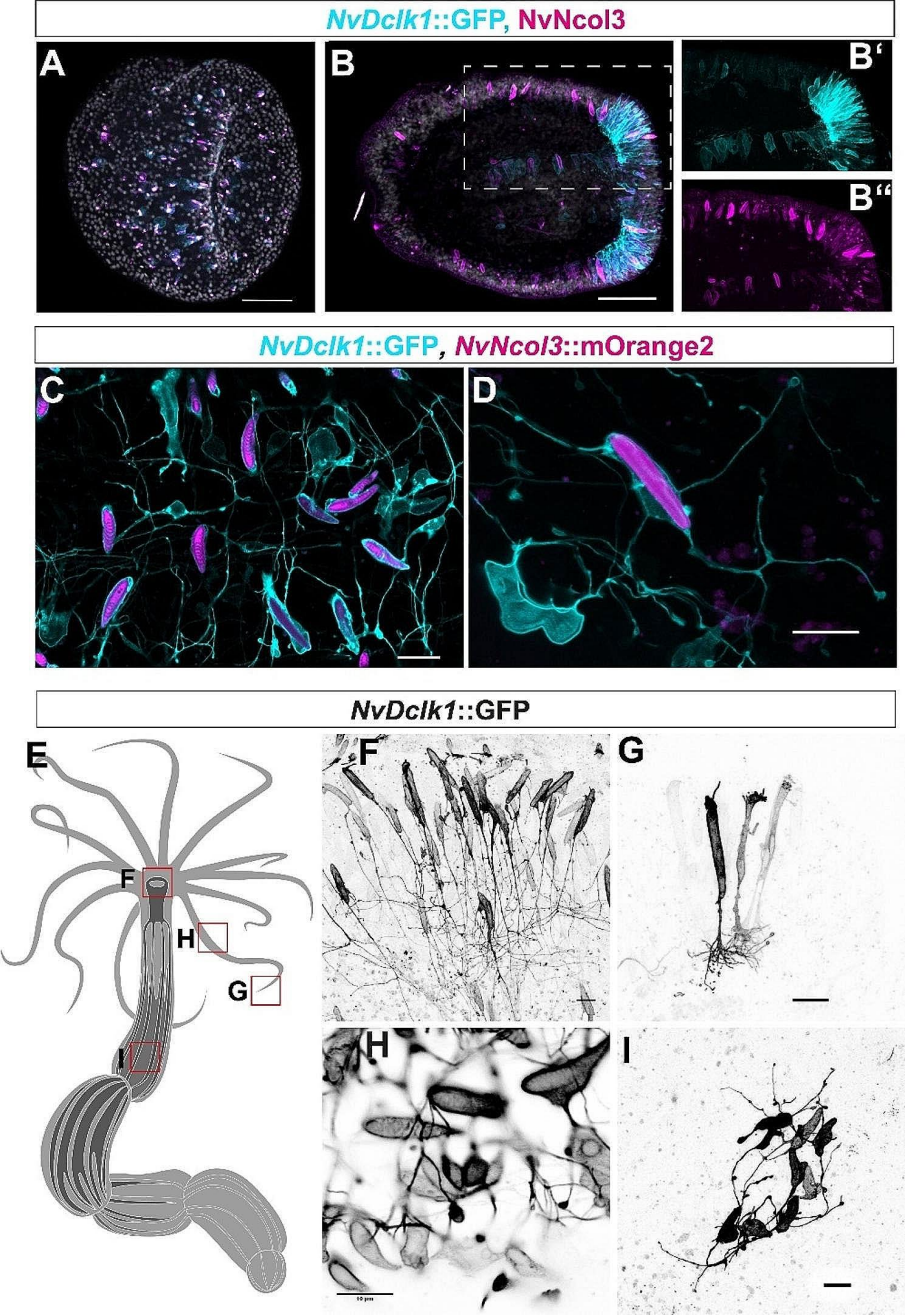
*NvDclk1*::GFP labels cnidocytes and their processes. (**A**, **B**) Lateral views of *NvDclk1*::GFP transgenic animals (cyan) co-labelled with anti-NvNcol3 antibody (magenta) at gastrula (**A**) and planula (**B**) stages. B’ and B’’ are single channel images of higher magnifications of the area boxed in (**B**), with DNA labelled by DAPI (grey). Many *NvDclk1*::GFP-positive cells express NvNcol3. (**C**, **D**) Cnidocytes in the tentacles of *NvDclk1*::GFP (shown in cyan), *NvNCol3*::mOrange2 (magenta) double transgenic animals possess long processes. (**E**-**I**) *NvDclk1*::GFP-expressing cnidocytes in different body regions display different morphologies. Images are from animals injected with the *NvDclk1::GFP* plasmid. A minimum of five animals was analysed for each of the indicated body regions. Scale bars: 50 μm (A, B), 10 μm (C-I)

To test whether *NvDclk1*::GFP labels cnidocytes, we labelled cnidocysts (the extrusive capsule inside cnidocytes) with an antibody against the minicollagen NvNCol3 [67]. Both at gastrula and planula stages, many of the GFP-positive cells also stained for NvNCol3 (Fig. 3A, B). At gastrula stage, many of the NvNCol3 - labelled cnidocysts were small (Fig. 3A), suggesting that the transgene identifies cnidocytes at an early stage in their development. At planula stage, *NvDclk1*::GFP-positive cnidocytes were visible both in the pharynx and in the tentacle buds (Fig. 3B). Crossing the *NvDclk1*::*GFP* animals to a *NvNCol3*::*mOrange2* line [65] showed that many of the labelled cnidocytes possess multiple long processes with bifurcations and varicosities, morphologically resembling neurites (Fig. 3C, D), as also described by Karabulut et al., 2022 [68] and partially visible in Weir et al., 2020 [69]. This network of cell processes was visible both in the tentacles and in the body column.

Observation of polyps mosaic for the *NvDclk1::GFP* transgene (i.e. F_0_ animals injected with the *NvDclk1::GFP* plasmid) identified differences in the morphology of labelled cells in different parts of the animals. Cnidocytes around the mouth opening and at the tentacle tips possessed long stalks connecting the cell body to the base of the epithelium, with the stalk branching into several shorter processes at the base (Fig. 3E-G). Cnidocytes with more proximal positions in the tentacle (closer to the mouth) and cells in the body column extended several processes, but they lacked prominent stalks towards the base of the epithelium (Fig. 3E, H, I).

**Fig. 4:**
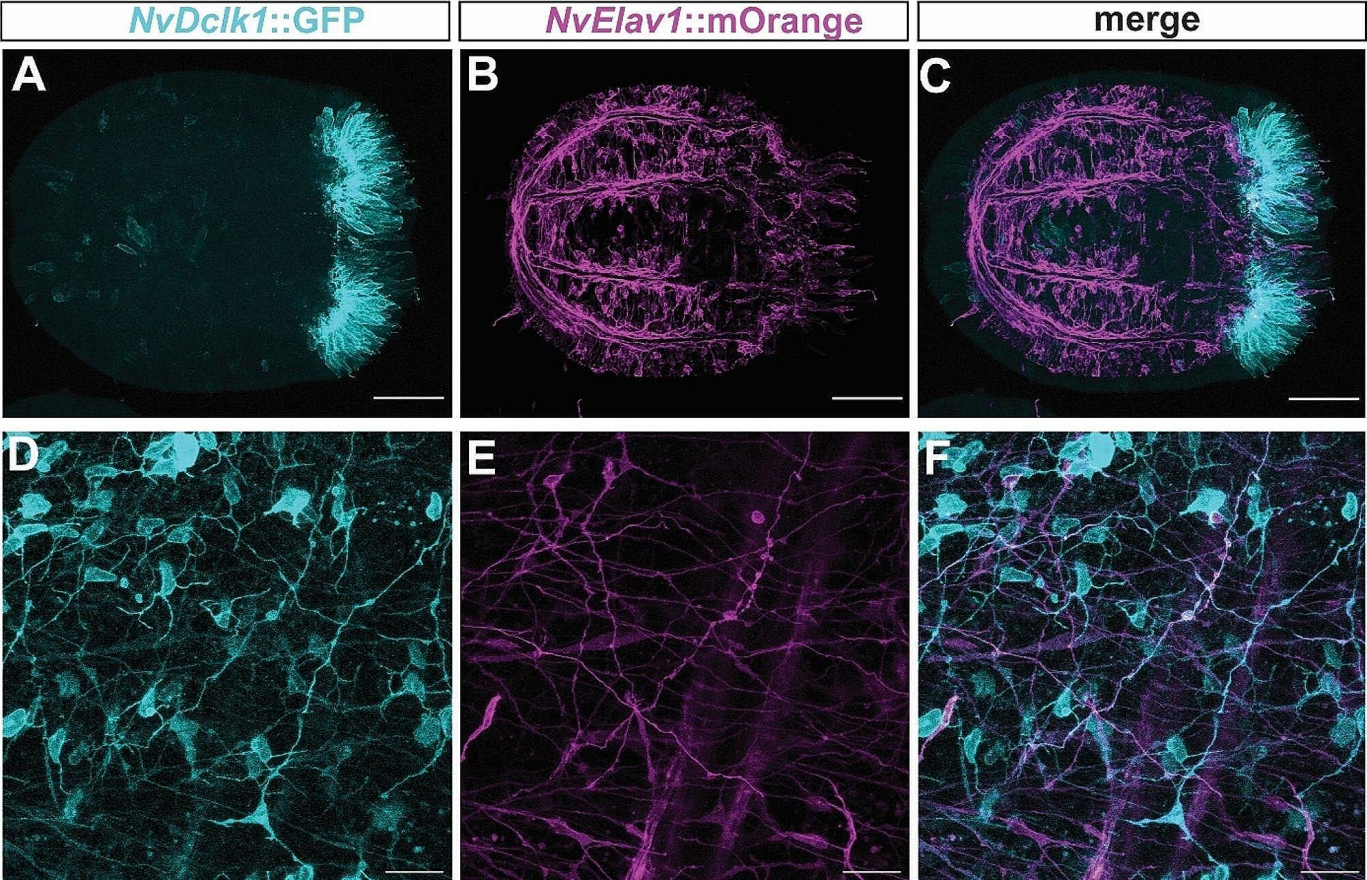
*NvDclk1*::GFP labels neurons in juvenile polyps. Confocal microscopy images of *NvDclk1*::GFP (in cyan), *NvElav1*::mOrange (in magenta) double transgenic animals. (**A**-**C**) Lateral views at tentacle bud stage, oral pole to the right. At this stage, co-expression of the two transgenes is not visible. (**D**-**F**) Views of the body column of 7 week-old polyps. Most *NvElav1*::mOrange-expressing neurons at this stage co-express *NvDclk1*::GFP. Two tracts of neurites along the base of the mesenteries running in an oblique orientation are visible. Scale bars: 50 μm (**A**-**C**), 20 μm (**D**-**F**)

Since *NvDclk1* transcripts are enriched in *NvElav1*::mOrange^+^ neurons, we next tested whether the *NvDclk1*::GFP transgene can be observed in these neurons. In double transgenic animals (with one allele of each transgene), GFP signal in the body column of planulae and primary polyps was weak, whereas the *NvElav1*::mOrange transgene labelled a large number of neurons and their neurites (Fig. 4A-C), as previously described [63]. In older polyps, starting at around six weeks of age, the *NvDclk1*::GFP transgene was visible in most (75.8 ± 10%, *n* = 3) of the *NvElav1*::mOrange-expressing neurons (Fig. 4D-F).

Taken together, the *NvDclk1*::GFP transgene labels cnidocytes that form an extensive network of neurite-like processes, and it is visible in a large part of the *NvElav1*::mOrange^+^ neurons of juvenile polyps.

### NvDclk1s localizes to microtubules in cnidocytes

**Fig. 5:**
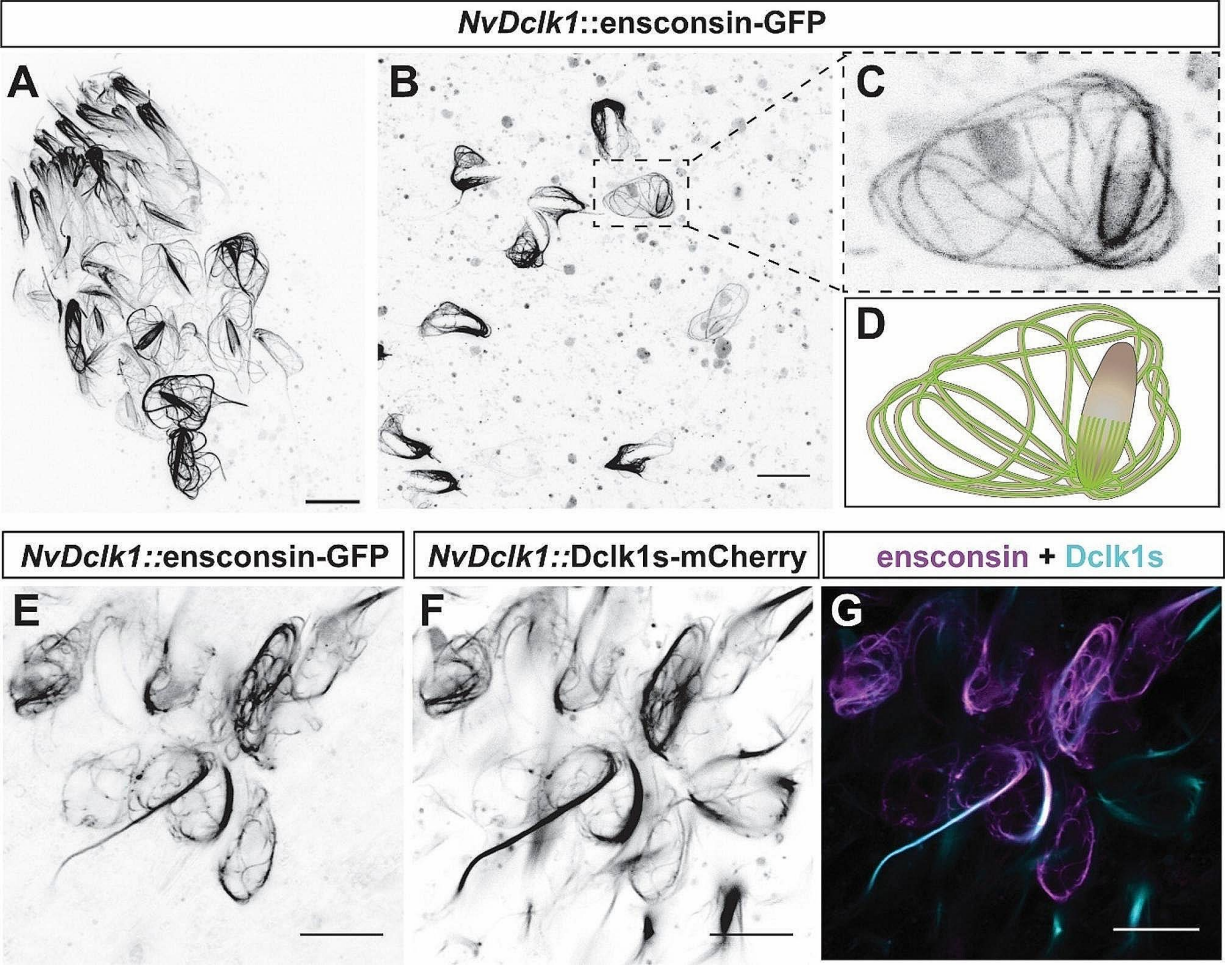
NvDclk1s localizes to microtubules in cnidocytes. Confocal images of live primary polyps injected with *NvDclk1::ensconsin-GFP* only (**A**-**C**) or together with *NvDclk1::Dclk1s-mCherry* (**E**-**G**). The injection results in patches of cells expressing the transgenes. (**A**) Tentacle tip, (**B**, **C**) body column of *NvDclk1*::ensconsin-GFP expressing polyps. (**C**) is a higher magnification of the area boxed in (**B**), (**D**) is an interpretation of (**C**) with cnidocyst capsule and thread in brown and the GFP signal in green. (**E**-**F**) body column of a polyp injected with both transgenes show co-localization of the two signals. Scale bars 10 μm

Dcx-domain containing proteins have been shown to bind to microtubules, and microtubules support the development of the cnidocyst in *Hydra* and other cnidarians [70–72]. To visualize the localization of microtubules in cnidocytes, we generated a transgene in which the regulatory elements of *NvDclk1* drive the expression of the microtubule-binding domain of human ensconsin fused to three copies of GFP (*NvDclk1*::ensconsin-GFP [73]) . Injection of this construct labelled patches of cells in varying areas of the animals. In the tentacle tips, the GFP signal was prominent in filamentous structures at the apical end of cnidocytes and in the cnidocil, the sensory structure protruding from the cnidocytes into the exterior (Fig. 5A). In the body column, we found cells with labelling of a convoluted thread-like structure connected to an oval capsule with more prominent GFP signal on one side of the long axis of the capsule (Fig. 5B, C). The non-invaginated thread suggests that these cells are immature cnidocytes and the observations with the *NvDclk1*::*ensconsin-GFP* transgene are in line with descriptions of microtubules in cnidocytes based on electron microscopy and immunohistochemistry [70–72]. To test whether NvDclk1 also localizes to microtubules in cnidocytes, we next generated a transgene in which a NvDclk1s-mCherry fusion protein is expressed under the control of the *NvDclk1* regulatory elements (*NvDclk1*::*Dclk1s-mCherry*). We injected this construct together with the *NvDclk1*::*ensconsin-GFP* construct to observe potential colocalization. Though we did not recover double positive patches in the tentacle tips, cells in the body column showed near complete colocalization of the ensconsin-GFP and Dclk1s-mCherry signals (Fig. 5E-G). This suggests that NvDclk1 localizes to microtubules and might function as a microtubule binding protein, as described for vertebrate Dclk proteins.

### ***NvDclk1*** mutants lack mature cnidocytes

To analyze the function of *NvDclk1* in cnidocyte development, we generated a mutant using the CRISPR/Cas9 system [74, 75]. The mutant has a 53 bp deletion located at the beginning of the first dcx domain. In the predicted protein resulting from this mutation, the first 14 amino acids of the first dcx domain are as in the wildtype allele, followed by 23 amino acids not found in the wildtype allele and a premature stop codon. The predicted protein is thus truncated before the middle of the first dcx domain and the mutation affects both splice variants (Additional Fig. 1A-C).

**Fig. 6:**
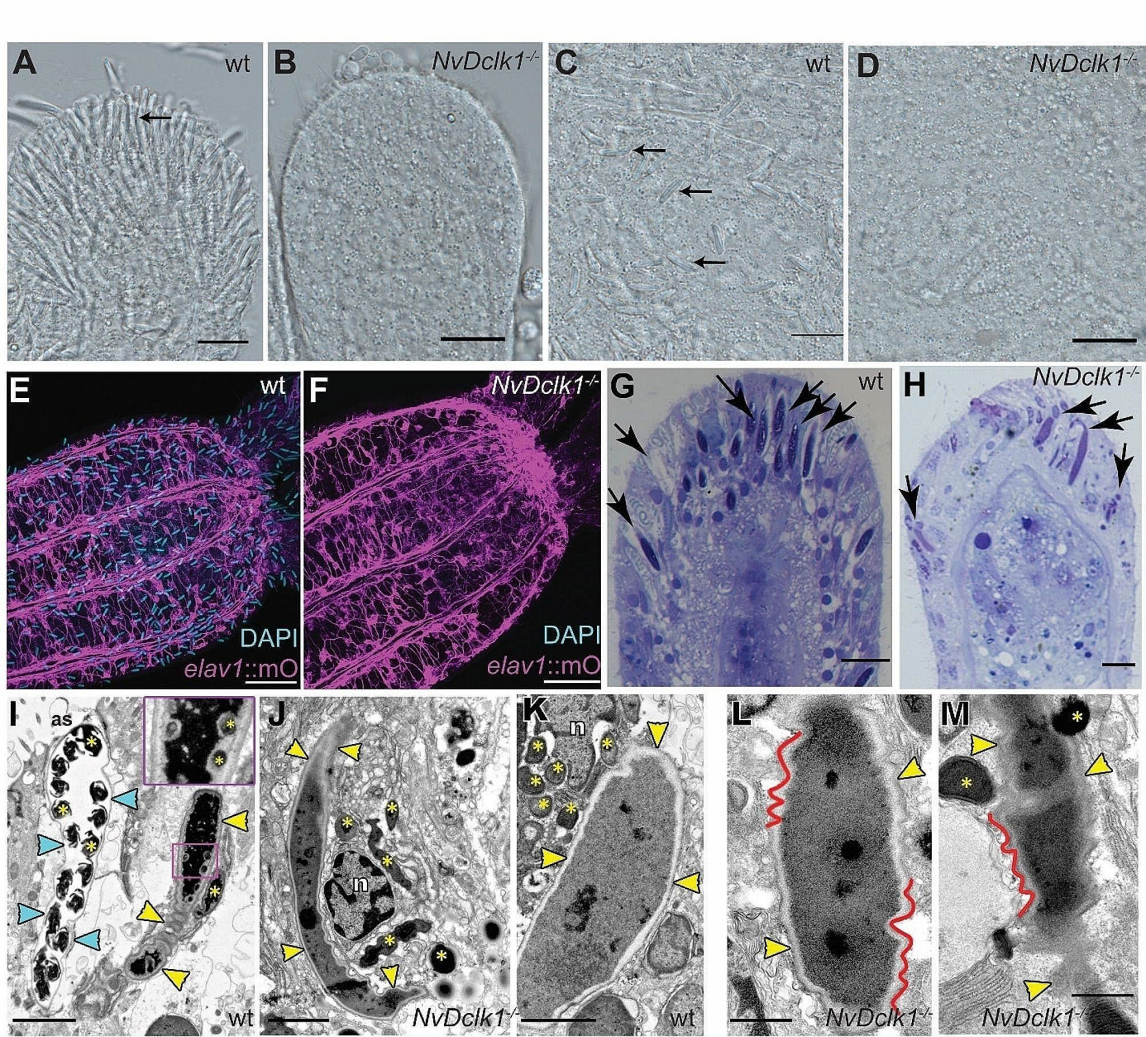
*NvDclk1* is required for cnidocyte differentiation. (**A**-**D**) DIC images of tentacle tips (**A**, **B**) and body colum (**C**, **D**) in the genotypes indicated in the upper right corner. Homozygous mutants lack the elongated cnidocysts that are found in the wildtype animals (arrows in **A**, **C**). (**E**, **F**) *NvElav1*::*mOrange* transgenics mutant for *NvDclk1* lack mature cnidocysts, as shown by the absence of DAPI signal. The *NvElav1*::mOrange-positive nervous system does not show gross alterations in the mutants. Lateral views with oral end oriented to the right/upper right. (**G**, **H**) Semi thin sections of tentacles show elongated cnidocytes in wildtype (arrows in **G**) and fewer cnidocytes with bent cnidocysts in *NvDclk1* mutants (arrow in **H**). *n* = 5 animals for wildtype, *n* = 16 animals for mutants. (**I**-**M**) Transmission electron microscopy of tentacle tips. In (**I**) cyan arrowheads indicate the cnidocyst of a mature, yellow arrowheads the capsule of an immature cnidocyte. The thread of the cnidocyst (yellow asterisks) is inside the mature cnidocyst, and partially outside the cnidocyst in the immature cnidocyst. In the inset in the upper right corner, yellow asterisks label invaginated parts of the thread. (**J**) TEM image of the cnidocyte in a *NvDclk1* mutant. The cnidocyte capsule is bent and the thread has not been invaginated (multiple thread fragments are marked with the asterisks). (**K**) The capsule of immature wt cnidocyte, multiple thread fragments located near the capsule are marked with the asterisks. Note that the capsule wall is almost even. (**L**, **M**) The capsules of cnidocytes typical for *NvDclk1* mutants; red lines follow parts of the uneven surface of the capsule walls. *n* = 3 animals for wild type, *n* = 5 animals for mutants. as – apical surface, n – cnidocyte nucleus. Scale bars 10 μm (A-D, G, H), 50 μm (E, F), 2 μm (I-K), 1 μm (L, M)

In crosses of heterozygous animals, we observed approximately 25% of primary polyps in which cnidocysts (the capsule inside the cnidocytes) were not visible in the tentacle tips (Fig. 6A, B) or the body cloumn (Fig. 6C, D) by light microscopy of living polyps. The cnidocyst phenotype in these animals was confirmed by a lack of signal from a modified DAPI staining protocol that labels the content of mature cnidocysts (Fig. 6E, F [76, 77]). PCR confirmed that these animals represent homozygous mutants for *NvDclk1* (Additional Fig. 1D). Histological staining of animals with this phenotype showed that a small number of cnidocyst-like structures were present in the tentacles, but they did not develop into the elongated capsules with an internalized thread that are observed in mature cnidocytes (Fig. 6G, H). Transmission electron microscopy revealed bent tentacle cnidocysts in the mutants that had not invaginated the thread (Fig. 6I, J) and often had an uneven surface of the capsule (Fig. 6K-M).

To analyze whether the nervous system is affected in *NvDclk1* mutants, we crossed the mutant allele into the *NvElav1*::*mOrange* line and selected offspring carrying both the transgene and one mutant allele. Incrossing of these animals (*NvDclk1*^*+/−*^, *NvElav1::mOrange*) resulted again in offspring that lacked cnidocysts in the tentacle tips, putatively homozygous for the mutant *NvDclk1* allele. In contrast to the absence of mature cnidocytes, we could not detect defects in the *NvElav1*::mOrange^+^ nervous system (Fig. 6E, F). We note, however, that subtle changes in the development or function of the nervous system would be difficult to detect with the available tools.

Taken together, the data show that *NvDclk1* is required for the development of cnidocytes, a derived neural cell type specific to cnidarians.

## Discussion

While many regulators of microtubules are present in all cell types of multicellular organisms, some regulate the cytoskeletal properties of only some cell types. Here we show that the microtubule-binding protein NvDclk1 is predominantly expressed in cells of the neural lineage in *Nematostella*, suggesting that the elaboration of neural morphology coincided with the acquisition of specific microtubule-regulating mechanisms during evolution. This is in line with a recent report in which comparative analyses of single cell RNA sequencing data suggested that genes related to neurite formation were gained in neural gene expression programs before the last common ancestor of cnidarians and bilaterians [78]. This study, however, further found that additional genes related to the organization of microtubules were added to these gene expression programs in the lineage leading to bilaterians [78].

Based on the previously described organization of microtubules during cnidocyte development, it is readily conceivable that perturbation of *NvDclk1* function interferes with cnidocyst formation. The cnidocyst is a large post-Golgi structure that forms by addition of smaller vesicles to the growing capsule. It has a tubular extension at one end that becomes part of the thread that will be extruded upon activation of the mature cnidocyst. The thread invaginates into the lumen of the capsule where it is further modified. Maturation of the cnidocyst also involves stiffening of the capsule wall to allow the generation of osmotic pressure inside the capsule [79, 80]. Microtubules have been visualized as a cage-like structure at the thread-bearing pole of the growing capsule and around the forming thread [70–72]. It has been proposed that they provide structural support for the growing thread and capsule, and they are potentially involved in the transport of vesicles from the Golgi to the capsule [70, 71]. Dclks and other dcx domain proteins have been shown to regulate the structure of the microtubule network and transport along microtubules [26, 29, 81]. Our observation that *NvDclk1* mutants contain cnidocysts with an irregular, bent shape and an uneven surface suggests that their maturation into elongated capsules with a smooth surface is perturbed. This is compatible with a role for *NvDclk1* in the formation or maintenance of a microtubule structure that provides structural support for the cnidocyst. It remains to be determined whether this is based on a direct role in regulating microtubule properties, a function in regulating microtubule-based transport, or both. Other dcx domain-containing proteins have been shown to have analogous roles in regulating microtubule-based structural support of organelles, e.g. for the nucleus in migrating neurons [17, 19] or for mechanosensory organelles in *Drosophila* mechanoreceptors [39, 82]. Though our data show that *NvDclk1* is required for the differentiation of cnidocytes, we currently cannot rule out that it has an additional, earlier role in their specification.

We have here identified two splice variants of *NvDclk1*, with *NvDclk1s* lacking the C-terminal kinase domain. Similar splice variants have been recovered for Dclk genes in mice [83, 84]. The *NvDclk1* mutant presented here truncates the protein in the first dcx domain. It thus does not distinguish between the long and short splice variants, and it therefore remains to be addressed whether the kinase domain is required for the function of *NvDclk1* in cnidocyte development.

While our observations suggest a role for *NvDclk1* in the regulation of microtubules, we currently cannot rule out that additional functions, e.g. in regulating the actin cytoskeleton [28, 30, 85], contribute to the phenotype of the *NvDclk1* mutants.

The *NvDclk1*::*GFP* transgenic line revealed an elaborate network of cellular processes emerging from cnidocytes, as was also shown with a *NvNematogalectin::EGFP* transgene in a recent paper [68]. It is tempting to speculate that these processes might represent neurites that allow communication among cnidocytes and between cnidocytes and other cells, e.g. neurons or secretory cells [86]. This possibility is further supported by electron microscopy reconstructions of cnidocytes in *Nematostella* [69] and by earlier observations of synaptic contacts between sensory cells and cnidocytes in other cnidarians [87, 88]. If cnidocytes engage in local circuitry controlling their activity, it will be interesting to understand how such circuitry remains functional, given that cnidocytes are thought to be single-use cells that are lost after extrusion of the cnidocyst.

In contrast to the readily visible defect in cnidocyte development, we did not observe an effect of the *NvDclk1* mutation on the structure of the *NvElav1*::mOrange-positive nervous system. In the double transgenics, co-expression of *NvDclk1*::GFP and *NvElav1*::mOrange was not visible at planula stages. Though this in principle could explain the lack of a phenotype at primary polyp stage, we rather think that the lack of observable co-expression is due to low-level expression of a single allele of the *NvDclk1*::*GFP* transgene at these stages. This scenario is in line with the finding that only crosses of two *NvDclk1*::*GFP* heterozygotes yielded visible GFP expression in the body column of planulae. The lack of an apparent phenotype might instead be due to functional redundancy with other microtubule-binding proteins in neurons, but we also acknowledge that we currently do not have the tools to identify subtle effects on neural development or structure, e.g. in the timing of neurite outgrowth, the polarity of microtubules in neurites [89] or the formation of synapses.

Taken together we show here that *NvDclk1* is predominantly expressed in cells of the *Nematostella* nervous system and that it is required for cnidocyte development. This supports the hypothesis that regulation of the microtubule cytoskeleton by dcx domain containing proteins is an ancient feature of nervous systems.

## Materials and methods

### Cloning

*NvDclk1s* was amplified based on EST cluster > 2663565_5 [90] with primers GGTTTTCAGTCCACCCGATCAG (forward) and GGATGAAAGACACTCCCAGTC (reverse). *NvDclk1l* was amplified based on gene models Nve15812 and Nve15813 (https://figshare.com/articles/Nematostella_vectensis_transcriptome_and_gene_models_v2_0/807696) with primers ATGGAGAATGGAATGATGAATGGAG (forward) and GACCCAGGGATGTGCGAGCAC (reverse). Both splice variants were cloned into pGEM-Teasy (Promega, US).

### Transgenic reporter lines

The *NvDclk1::GFP* and *NvDclk1::dclk1s-mCherry* lines were generated by I-SceI-mediated transgenesis as described previously [91, 92]. For the *NvDclk1* reporter line ca. 2.9 kb upstream of the start codon were amplified and inserted via PacI and AscI sites in front of a GFP sequence with a C-terminal CAAX motif for membrane localization. The genomic coordinates for the regulatory region are 13,242,493–13,239,561 on the minus strand of chromosome 10 (https://simrbase.stowers.org/). For the *NvDclk1::dclk1s-mCherry* line, the ORF of *NvDclk1s* was inserted with AscI/AscI between the *NvDclk1* promoter and the ORF for mCherry. For *NvDclk1::ensconsin-GFP*, the microtubule-binding domain of human ensconsin fused to three copies of EGFP [73] was amplified from Addgene plasmid #26,741 and inserted with PacI/AscI downstream of the *NvDclk1* promoter. In all constructs, the cloning cassette was flanked by inverted I-SceI sites. Plasmids were digested with I-SceI for 30 min at 37ºC and injected at a concentration of 20 μg/μl [91, 93].

### Generation and genotyping of NvDclk1 mutants

The mutants were generated following the protocol described in [94]. Single guide RNAs targeting the start of the first dcx domain were synthesized using primers TAGGTTTCAAGCGAGCGCTACA and AAACTGTAGCGCTCGCTTGAAA. Primers were annealed and inserted into vector pDR274 [94]. sgRNAs were synthesized using the T7 MegaScript kit (Invitrogen, USA). 500 ng/μl single guide RNA and 1.5 μg/μl nls-Cas9 (PNAbio, USA) were injected into fertilized eggs. Foot tissue of F1 animals was genotyped by amplifying and sequencing the region around the sgRNA target site with primers GCATCGGGAGATAGAATGGA (forward) and GAAGGTCGATTTTCGCAGAC (reverse). Polyps heterozygous for a 53 bp deletion were crossed to obtain homozygous F2 and F3 mutants. The genotype of polyps lacking visible tentacle cnidocysts was determined by PCR with the same primers.

### In situ hybridization and immunohistochemistry

Colorimetric and fluorescent in situ hybridization were done according to the protocols described in [59] and [61]. The following protocol is edited from [61].

Embryos were fixed in 3.7% formaldehyde/0.25% glutaraldehyde/NM for 2 min on ice, then in 3.7% formaldehyde/PTW (PBS + 0.1%Tween20) for 1 h at 4 C. For colorimetric in situ hybridization, samples were rehydrated in PTW, incubated in 20 mg/ml Proteinase K for 10 min at room temperature (RT) followed by washes in 4 mg/ml Glycine/PTW. They were then washed in 1% triethanolamine in PBS, followed by the addition of 0.25%, then 0.5% acetic anhydride. Samples were washed in PTW and refixed in 3.7% formaldehyde/PTW, followed by washes in PTW. Pre-hybridization in hybridization buffer (HB: 50% formamide, 5X SSC, 1% SDS, 50 mg/ml heparin, 100 mg/ml salmon sperm DNA, 9.25 mM citric acid, 0.1X Tween20) was for at least 2 h at 60ºC. Digoxigenin-labeled riboprobes were synthesized from PCR templates (MEGAscript Kit, Invitrogen) and incubated with the samples for at least 60 h at 60ºC. Unbound probe was removed via a series of 60ºC washes of HB/2X SSC solutions [75/25, 50/50, 25/75, 0/100 (v/v)], then 0.2X SSC, 0.1XSSC. This was followed by RT washes of SSC/PTW solutions [75/25, 50/50, 25/75, 0/100 (v/v)]. Samples were then blocked in blocking solution [1% Block (Roche)/Maleic acid buffer (100mM maleic acid, 150mM NaCl)] for 2 h at RT and incubated overnight with 1:5000 anti-digoxigenin alkaline phosphatase (Roche)/blocking solution. Unbound antibody was removed with 10 × 15 min washes of PBTxBSA (PBS /0.2%TritonX-100 /0.1% BSA); samples were then washed with staining buffer (100mM Tris pH 9.5, 100mM NaCl, 50mM MgCl_2_, 0.1%Tween20) before colour was initiated by the addition of 1:200 NBT/BCIP solution (Roche). To stop the reaction, samples were washed in staining buffer, PTW, H_2_0, ethanol, H_2_0, PTW and then post-fixed for 30 min with 3.7% formaldehyde/PTW before being washed with PTW and cleared by overnight incubation in 87% glycerol at 4ºC.

For fluorescence in situ hybridization, fixed samples were incubated in 2% hydrogen peroxide in methanol to quench endogenous hydrogen peroxidase activity. Samples were then rehydrated in PTW and the ISH protocol was followed from the Proteinase K incubation step until the end of the SSC/PTW RT washes. Samples were incubated with digoxigenin and fluorescein-labeled riboprobes (MEGAscript Kit, Invitrogen). After the SSC/PTW RT washes, samples were washed in TNT (0.1 M Tris-HCl pH 7.5/0.15 M NaCl/0.5% Triton X-100) and then blocked in TNTblock [0.5% blocking reagent (PerkinElmer)/TNT] for 1 h at RT before overnight incubation with anti-digoxigenin (1:100) or anti-fluorescein (1:250) horseradish peroxidase (Roche). Unbound antibodies were removed by 10 15 min TNT washes, and samples were then incubated in fluorophore tyramide amplification reagent (TSA Plus Kit, Perkin Elmer). For double labeling, samples were washed in 0.1 M glycine pH 2.0 and incubated 1 h in TNT block before overnight incubation with anti-digoxygenin or anti-fluorescein horseradish peroxidase (Roche). Post-antibody washing and the TSA reaction were repeated as for the first probe; samples were then washed in TNT, incubated with DAPI 1:1000 and mounted in ProLong Gold antifade reagent.

Samples were imaged on either a Nikon Eclipse E800 compound microscope with a Nikon Digital Sight DSU3 camera, on a Leica SP5 or an Olympus FV3000 confocal microscope.

For immunohistochemistry, fixed samples were washed for 2 h with PBTx (PBS/0.3%Triton X-100) and then incubated in block (5% normal goat serum/PBTx) for 1 h at RT before overnight incubation in primary antibodies at 4 C. Samples were then washed for 2 h with PBTx, incubated for 1 h at RT in block and then overnight at 4ºC in Alexa Fluor conjugated secondary antibodies (Molecular Probes, 1:200). Samples were then washed for 2 h with PBTx, incubated for 30 min in DAPI 1:1000 (Molecular Probes) and mounted in ProLong Gold antifade reagent (Molecular Probes). The following primary antibodies were used: to detect GFP, anti-GFP (mouse, abcam1218, 1:200); to detect mOrange or mCherry, anti dsRed (rabbit, Clontech 632,496, 1:100); anti-NCol3 (Zenkert et al., 2011). Mature cnidocytes were labeled with DAPI/EDTA (Fig. 6) as described in [80] and [76]. For Fig. 5, polyps were imaged live on a Leica SP5 confocal microscope.

### Histology and electron microscopy

For histology and electron microscopy, primary polyps were fixed in 2.5% glutaraldehyde/0.1 M cacodylate buffer (pH 7.2). Samples were transferred into 1.25% glutaraldehyde/0.1 M cacodylate buffer (pH 7.2) and stored at 4 °C until further processing. Polyps were then washed in 0.1 M cacodylate buffer and postfixed in 1% osmium tetroxide in the same buffer for 1 h. Samples were dehydrated through a graded series of ethanol and acetone and then embedded into Epon resine (SPI-Pon 812 Embedding Kit, Structure Probe Inc.) and sectioned using routine techniques by Leica ULTRACUT microtome. Semithin sections were stained in 1% solution of toluidine blue. Ultrathin sections were stained in 2% water solutions of uranyl acetate and in Reynolds’ lead citrate solution [95] and examined by transmission electron microscopes JEOL JEM-1011 and JEOL JEM-1400. Sample processing was performed at the Michael Sars Centre (Bergen) and electron microscopy was performed at the Electron Microscopy Laboratory of the Shared Facilities Center of the Lomonosov Moscow State University.

### Electronic supplementary material

Below is the link to the electronic supplementary material.

Supplementary Material 1

## Data Availability

No datasets were generated or analysed during the current study.

## References

[1] Luo L (2002). Actin cytoskeleton regulation in neuronal morphogenesis and structural plasticity. Annu Rev Cell Dev Biol.

[2] Campellone KG, Welch MD (2010). A nucleator arms race: cellular control of actin assembly. Nat Rev Mol Cell Biol.

[3] Coles CH, Bradke F (2015). Coordinating neuronal actin-microtubule dynamics. Curr Biol.

[4] Laser-Azogui A (2015). Neurofilament assembly and function during neuronal development. Curr Opin Cell Biol.

[5] Janke C (2014). The tubulin code: molecular components, readout mechanisms, and functions. J Cell Biol.

[6] Goodson HV, Jonasson EM. Microtubules and microtubule-associated proteins. Cold Spring Harb Perspect Biol. 2018;10(6). 10.1101/cshperspect.a022608 PMC5983186 29858272

[7] Conde C, Caceres A (2009). Microtubule assembly, organization and dynamics in axons and dendrites. Nat Rev Neurosci.

[8] Kapitein LC, Hoogenraad CC (2015). Building the neuronal Microtubule Cytoskeleton. Neuron.

[9] Reiner O (2006). The evolving doublecortin (DCX) superfamily. BMC Genomics.

[10] Coquelle FM (2006). Common and divergent roles for members of the mouse DCX superfamily. Cell Cycle.

[11] Dijkmans TF (2010). The doublecortin gene family and disorders of neuronal structure. Cent Nerv Syst Agents Med Chem.

[12] des Portes V (1998). A novel CNS gene required for neuronal migration and involved in X-linked subcortical laminar heterotopia and lissencephaly syndrome. Cell.

[13] Gleeson JG (1998). Doublecortin, a brain-specific gene mutated in human X-linked lissencephaly and double cortex syndrome, encodes a putative signaling protein. Cell.

[14] Francis F (1999). Doublecortin is a developmentally regulated, microtubule-associated protein expressed in migrating and differentiating neurons. Neuron.

[15] Gleeson JG (1999). Doublecortin is a microtubule-associated protein and is expressed widely by migrating neurons. Neuron.

[16] Corbo JC (2002). Doublecortin is required in mice for lamination of the hippocampus but not the neocortex. J Neurosci.

[17] Koizumi H, Tanaka T, Gleeson JG (2006). Doublecortin-like kinase functions with doublecortin to mediate fiber tract decussation and neuronal migration. Neuron.

[18] Deuel TA (2006). Genetic interactions between doublecortin and doublecortin-like kinase in neuronal migration and axon outgrowth. Neuron.

[19] Tanaka T, Koizumi H, Gleeson JG (2006). The doublecortin and doublecortin-like kinase 1 genes cooperate in murine hippocampal development. Cereb Cortex.

[20] Jean DC, Baas PW, Black MM (2012). A novel role for doublecortin and doublecortin-like kinase in regulating growth cone microtubules. Hum Mol Genet.

[21] Koizumi H (2017). DCLK1 phosphorylates the microtubule-associated protein MAP7D1 to promote axon elongation in cortical neurons. Dev Neurobiol.

[22] Shin E (2013). Doublecortin-like kinase enhances dendritic remodelling and negatively regulates synapse maturation. Nat Commun.

[23] Burgess HA, Reiner O (2000). Doublecortin-like kinase is associated with microtubules in neuronal growth cones. Mol Cell Neurosci.

[24] Tanaka T (2004). Lis1 and doublecortin function with dynein to mediate coupling of the nucleus to the centrosome in neuronal migration. J Cell Biol.

[25] Liu JS (2012). Molecular basis for specific regulation of neuronal kinesin-3 motors by doublecortin family proteins. Mol Cell.

[26] Lipka J (2016). Microtubule-binding protein doublecortin-like kinase 1 (DCLK1) guides kinesin-3-mediated cargo transport to dendrites. EMBO J.

[27] Lin PT (2000). DCAMKL1 encodes a protein kinase with homology to doublecortin that regulates microtubule polymerization. J Neurosci.

[28] Nawabi H (2015). Doublecortin-Like Kinases promote neuronal survival and induce growth cone reformation via distinct mechanisms. Neuron.

[29] Fu X et al. Doublecortin and JIP3 are neural-specific counteracting regulators of dynein-mediated retrograde trafficking. Elife. 2022;11. 10.7554/eLife.82218 PMC9799976 36476638

[30] Fu X (2013). Doublecortin (Dcx) family proteins regulate filamentous actin structure in developing neurons. J Neurosci.

[31] Tsukada M (2005). Doublecortin association with actin filaments is regulated by neurabin II. J Biol Chem.

[32] Tint I (2009). Doublecortin associates with microtubules preferentially in regions of the axon displaying actin-rich protrusive structures. J Neurosci.

[33] Bourgeois F (2015). A critical and previously unsuspected role for doublecortin at the neuromuscular junction in mouse and human. Neuromuscul Disord.

[34] Ogawa R (2015). Doublecortin marks a new population of transiently amplifying muscle progenitor cells and is required for myofiber maturation during skeletal muscle regeneration. Development.

[35] Chhetri D (2022). Pleiotropic effects of DCLK1 in cancer and cancer stem cells. Front Mol Biosci.

[36] Nakanishi Y (2013). Dclk1 distinguishes between tumor and normal stem cells in the intestine. Nat Genet.

[37] Meyer I, Kuhnert O, Graf R (2011). Functional analyses of lissencephaly-related proteins in Dictyostelium. Semin Cell Dev Biol.

[38] Bechstedt S (2010). A doublecortin containing microtubule-associated protein is implicated in mechanotransduction in Drosophila sensory cilia. Nat Commun.

[39] Song X et al. DCX-EMAP is a core organizer for the ultrastructure of *Drosophila* mechanosensory organelles. J Cell Biol. 2023;222(10). 10.1083/jcb.202209116 PMC10471123 37651176

[40] Sopko R (2014). Combining genetic perturbations and proteomics to examine kinase-phosphatase networks in Drosophila embryos. Dev Cell.

[41] Gonczy P (2001). zyg-8, a gene required for spindle positioning in C. Elegans, encodes a doublecortin-related kinase that promotes microtubule assembly. Dev Cell.

[42] Bellanger JM (2012). The doublecortin-related gene zyg-8 is a microtubule organizer in Caenorhabditis elegans neurons. J Cell Sci.

[43] Xu D (2021). Functional contribution of DCLKs in sea urchin development. Dev Dyn.

[44] Telford MJ, Budd GE, Philippe H (2015). Phylogenomic insights into animal evolution. Curr Biol.

[45] Dunn CW et al. Animal phylogeny and its evolutionary implications. Annu Rev Ecol Evol S. 2014;45:371–395.

[46] dos Reis M (2015). Uncertainty in the timing of origin of animals and the limits of Precision in Molecular timescales. Curr Biol.

[47] Hand C, Uhlinger K (1992). The culture, sexual and asexual reproduction, and growth of the sea anemone Nematostella Vectensis. Biol Bull.

[48] Layden MJ, Rentzsch F, Rottinger E (2016). The rise of the starlet sea anemone Nematostella Vectensis as a model system to investigate development and regeneration. WIREs Dev Biol.

[49] Magie CR, Daly M, Martindale MQ (2007). Gastrulation in the cnidarian Nematostella vectensis occurs via invagination not ingression. Dev Biol.

[50] Kraus Y, Technau U (2006). Gastrulation in the sea anemone Nematostella vectensis occurs by invagination and immigration: an ultrastructural study. Dev Genes Evol.

[51] Galliot B (2009). Origins of neurogenesis, a cnidarian view. Dev Biol.

[52] Watanabe H, Fujisawa T, Holstein TW (2009). Cnidarians and the evolutionary origin of the nervous system. Dev Growth Differ.

[53] Rentzsch F, Layden M, Manuel M. The cellular and molecular basis of cnidarian neurogenesis. Wiley Interdiscip Rev Dev Biol. 2017;6(1). 10.1002/wdev.257 PMC6680159 27882698

[54] Siebert S et al. Stem cell differentiation trajectories in *Hydra* resolved at single-cell resolution. Science. 2019;365(6451). 10.1126/science.aav9314 PMC7104783 31346039

[55] Tourniere O (2022). Insm1-expressing neurons and secretory cells develop from a common pool of progenitors in the sea anemone Nematostella Vectensis. Sci Adv.

[56] Steger J (2022). Single-cell transcriptomics identifies conserved regulators of neuroglandular lineages. Cell Rep.

[57] Layden MJ, Boekhout M, Martindale MQ (2012). Nematostella vectensis achaete-scute homolog NvashA regulates embryonic ectodermal neurogenesis and represents an ancient component of the metazoan neural specification pathway. Development.

[58] Layden MJ, Martindale MQ (2014). Non-canonical notch signaling represents an ancestral mechanism to regulate neural differentiation. Evodevo.

[59] Richards GS, Rentzsch F (2014). Transgenic analysis of a SoxB gene reveals neural progenitor cells in the cnidarian Nematostella vectensis. Development.

[60] Richards GS, Rentzsch F (2015). Regulation of Nematostella neural progenitors by SoxB, notch and bHLH genes. Development.

[61] Tourniere O (2020). NvPOU4/Brain3 functions as a terminal selector gene in the nervous system of the Cnidarian Nematostella vectensis. Cell Rep.

[62] Watanabe H (2014). Sequential actions of beta-catenin and bmp pattern the oral nerve net in Nematostella vectensis. Nat Commun.

[63] Nakanishi N (2012). Nervous systems of the sea anemone Nematostella vectensis are generated by ectoderm and endoderm and shaped by distinct mechanisms. Development.

[64] Venkat A et al. Mechanistic and evolutionary insights into isoform-specific ‘supercharging’ in DCLK family kinases. Elife. 2023;12. 10.7554/eLife.87958 PMC10602587 37883155

[65] Sunagar K (2018). Cell type-specific expression profiling unravels the development and evolution of stinging cells in sea anemone. BMC Biol.

[66] Gahan JM (2022). Histone demethylase Lsd1 is required for the differentiation of neural cells in Nematostella vectensis. Nat Commun.

[67] Zenkert C (2011). Morphological and molecular analysis of the Nematostella vectensis cnidom. PLoS ONE.

[68] Karabulut A (2022). The architecture and operating mechanism of a cnidarian stinging organelle. Nat Commun.

[69] Weir K et al. A molecular filter for the cnidarian stinging response. Elife. 2020;9. 10.7554/eLife.57578 PMC7250568 32452384

[70] Holstein T (1981). The morphogenesis of nematocytes in Hydra and Forskalia: an ultrastructural study. J Ultrastruct Res.

[71] Engel U (2002). Nowa, a novel protein with minicollagen cys-rich domains, is involved in nematocyst formation in. J Cell Sci.

[72] Golz R (1994). Apical surface of Hydrozoan Nematocytes - Structural adaptations to Mechanosensory and exocytotic functions. J Morphol.

[73] Miller AL, Bement WM (2009). Regulation of cytokinesis by rho GTPase flux. Nat Cell Biol.

[74] Ikmi A (2014). TALEN and CRISPR/Cas9-mediated genome editing in the early-branching metazoan Nematostella vectensis. Nat Commun.

[75] Kraus Y (2016). Pre-bilaterian origin of the blastoporal axial organizer. Nat Commun.

[76] Szczepanek S, Cikala M, David CN (2002). Poly-gamma-glutamate synthesis during formation of nematocyst capsules in Hydra. J Cell Sci.

[77] Marlow HQ (2009). Anatomy and development of the nervous system of Nematostella vectensis, an anthozoan cnidarian. Dev Neurobiol.

[78] Najle SR (2023). Stepwise emergence of the neuronal gene expression program in early animal evolution. Cell.

[79] Beckmann A, Ozbek S (2012). The nematocyst: a molecular map of the cnidarian stinging organelle. Int J Dev Biol.

[80] Babonis LS, Martindale MQ (2017). PaxA, but not PaxC, is required for cnidocyte development in the sea anemone Nematostella Vectensis. Evodevo.

[81] Moores CA (2004). Mechanism of microtubule stabilization by doublecortin. Mol Cell.

[82] Liang X, Madrid J, Howard J (2014). The microtubule-based cytoskeleton is a component of a mechanical signaling pathway in fly campaniform receptors. Biophys J.

[83] Bergoglio E (2021). Spatial and temporal diversity of DCLK1 isoforms in developing mouse brain. Neurosci Res.

[84] Burgess HA, Reiner O (2002). Alternative splice variants of doublecortin-like kinase are differentially expressed and have different kinase activities. J Biol Chem.

[85] Garg N (2023). Non-muscle myosin II drives critical steps of nematocyst morphogenesis. iScience.

[86] Moran Y (2012). Neurotoxin localization to ectodermal gland cells uncovers an alternative mechanism of venom delivery in sea anemones. Proc Biol Sci.

[87] Westfall JA (2004). Neural pathways and innervation of cnidocytes in tentacles of sea anemones. Hydrobiologia.

[88] Westfall JA, Landers DD, McCallum JD (1999). Ultrastructure of neuro-spirocyte synapses in the sea anemone Aiptasia Pallida (Cnidaria, Anthozoa, Zoantharia). J Morphol.

[89] Stone MC et al. Cytoskeletal and synaptic polarity of LWamide-like + ganglion neurons in the sea anemone *Nematostella vectensis.* J Exp Biol. 2020;223(Pt 21). 10.1242/jeb.233197 PMC7673360 32968001

[90] Technau U (2005). Maintenance of ancestral complexity and non-metazoan genes in two basal cnidarians. Trends Genet.

[91] Renfer E (2009). A muscle-specific transgenic reporter line of the sea anemone, Nematostella vectensis. Proc Natl Acad Sci U S A.

[92] Renfer E, Technau U (2017). Meganuclease-assisted generation of stable transgenics in the sea anemone Nematostella Vectensis. Nat Protoc.

[93] Rentzsch F, Renfer E, Technau U (2020). Generating Transgenic reporter lines for studying nervous System Development in the Cnidarian Nematostella vectensis. Methods Mol Biol.

[94] Hwang WY (2013). Efficient genome editing in zebrafish using a CRISPR-Cas system. Nat Biotechnol.

[95] Reynolds ES (1963). The use of lead citrate at high pH as an electron-opaque stain in electron microscopy. J Cell Biol.

