# Supplementary material for: Doublecortin-like kinase is required for cnidocyte development in Nematostella vectensis
Source: Neural Dev. 2024 Jun 22;19:11. doi: 10.1186/s13064-024-00188-0 (PMC11193195; doi:10.1186/s13064-024-00188-0)
Supplement: Supplementary file 1 — Supplementary Material 1 [file 13064_2024_188_MOESM1_ESM.pdf]

Supplementary Material for

Doublecortin-like kinase is required for cnidocyte development in *Nematostella vectensis*

Johanna E. M. Kraus, Henriette Busengdal, Yulia Kraus, Harald Hausen, Fabian Rentzsch

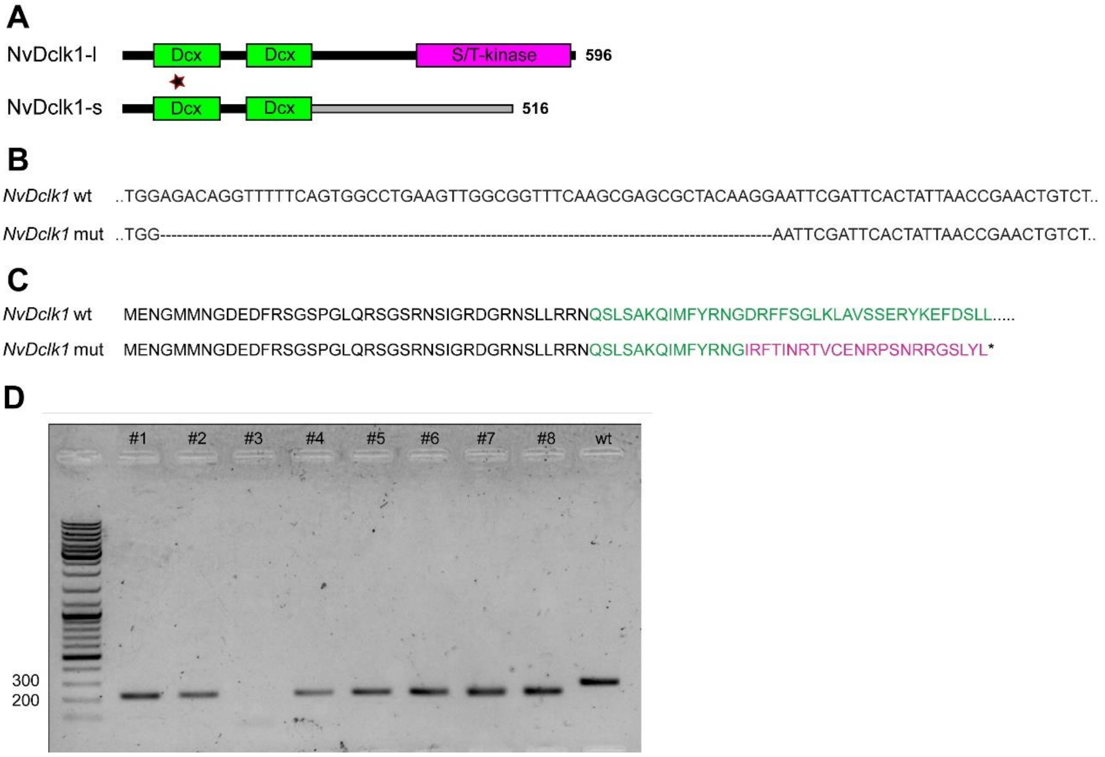

Additional Figure 1: Genotype of the *NvDclk1* mutant. (A) Cartoon of the two *NvDclk1* splice variants, the star indicates the approximate site of the premature stop codon. (B) Nucleotide sequences of the wildtype and mutant alleles. (C) Amino acid sequences of the wildtype and mutant alleles at the N-terminus. The dcx domain is highlighted in green, the sequence only present only in the mutant allele in magenta. (D) Agarose gel showing amplifications of the mutated site in eight primary polyps that lacked visually recognizable cnidocysts in the tentacles (#1-8, example shown in Figure 6B) and one wildtype primary polyp. For #3, genomic DNA isolation failed.
